# Supplementary material for: Enhancing coevolution-based contact prediction by imposing structural self-consistency of the contacts
Source: Sci Rep. 2018 Jul 24;8:11112. doi: 10.1038/s41598-018-29357-y (PMC6057941; doi:10.1038/s41598-018-29357-y)
Supplement: Supplementary file 1 — Supporting information [file 41598_2018_29357_MOESM1_ESM.pdf]

Enhancing coevolution-based contact prediction by imposing structural self-consistency of the contacts

Maher M. Kassem, Lars B. Christoffersen, Andrea Cavalli, Kresten Lindorff-Larsen

Other coevolution contact predictors

To ensure that CE-YAPP generalizes across different contact predictors, we applied CE-YAPP on multiple contact predictors. We included plmDCA [1, 2], which, exactly like Gremlin, uses a pseudo-likelihood maximization approach and should be very similar to Gremlin in its predictions. The other methods are, GaussDCA (gDCA [3]), CMAT [4] and PconsC3 [3, 5]. It should be noted, that PconsC3 uses a machine learning approach that combines plmDCA, gDCA and RaptorX [6], for which the latter provides a webserver that we cannot provide the input multiple sequence alignment to. Since, this is a requirement for us, to prevent the selection bias, observed when using too rich multiple sequence alignments, we chose to omit RaptorX and use CMAT as a replacement. We acknowledge that PconsC3 likely underperforms due to this replacement.

We predicted contacts for the Noumenon data set using the above-described methods, and performed contact filtering using CE-YAPP. The precision of the predicted contacts before and after applying CE-YAPP are shown in Fig. S2. Apart from CMAT, the different contact prediction methods perform similarly in terms of precision with CE-YAPP increasing the precision

similarly, on the Noumenon data set.

CE-YAPP parameter selection

Several parameters, in the CE-YAPP method, were either manually tuned or chosen based on previous work [7]. The parameters in question are (I) the number of input contacts, N_{input}, (II) the number of time steps during structure calculations, N_{steps}, (III) the number of repeated simulations, $N_{repeats}$, and (IV) D and d^0 (Eq. 2 in main text).

CE-YAPP was generally robust to changes in the following parameters: $N_{repeats}$, N_{steps}, D and d^0. The values assigned to these parameters were tuned based on the structural accuracy ($C\alpha$-RMSD) obtained when running CE-YAPP on three proteins with PDB IDs: 2RQL (95 amino acids), 5P21 (166 amino acids) and 1SVN (269 amino acids). We varied $N_{repeats}$ and N_{steps} individually, while D and D^0 were varied combinatorially.

Based on the results depicted in Fig. S3, we chose $N_{steps} = 10,000$, $N_{repeats} = 64$, $D = 3$ and $d^0 = 7$. Indeed, for these values, the structural accuracy was maximal for the three proteins.

Number of input contacts

To select the number of input contacts, we varied X in

$$N_{input} = X \times N_{AA} \tag{1}$$

where N_{AA} is the number of amino acids. We maximized for the total mean structural accuracy across 16 repeated simulations and each of the proteins in the Noumenon data set. In this case, we used the global distance test (GDT) with a single cutoff of 5 Å. In other words, we used a "low resolution" structural accuracy measure that reports on the ratio of $C\alpha$ atoms that are within 5 Å of the experimental PDB structure. As seen in Fig. S4, there is a peak at $X = 1.2$, which we chose as a final parameter. It should be noted that CE-YAPP is fairly robust to changes in X, with a mean increase of ~ 0.04 GDT(5) going from $X = 0.5$ to $X = 1.2$.

Equilibrium Simulations

In order to examine the dynamics of λ_i (Eq. 2 & 3, main text) in an equilibrium framework, we performed Monte Carlo simulations of a 20 amino acid long peptide called GSGS which natively forms a three-stranded anti-parallel β-sheet. We manually selected two true Cβ-Cβ contacts between each interface of strands, and a single false contact from strand one to strand three (Fig. S5a). We fixed the secondary structure to canonical extended strands according to the secondary structure labels shown in Fig. S5b. In this example, the only amino acids with dihedral angles that can change during simulation, are the two terminal residues and the two pairs of linker residues between neighbouring strands. For each Monte Carlo step, we randomly change the dihedrals of a single (allowed) residue and a random λ value, and accept or reject based on the Metropolis-Hastings criterion using the full en-

ergy function (Eq. 1, main text). The values of d^0 and D (Eq. 2, main text), were set to 7.0 Å and 1 Å, respectively. In order to get converged statistics, we selected a simulation temperature, $T = 2$, for which the protein would unfold and refold in a single simulation.

During the simulations, we monitored the structural RMSD with respect to the native structure as well as the five λ-values (Fig. S6). We find that the λ-values for true contacts (Fig. S6b and c) are highly dynamic; in contrast the λ-value for the false contact remains close to zero (Fig. S6d). We observe a correlation between λ-values for the contacts between strand 1 and 2 and the structural RMSD. Indeed, when the RMSD is large (≈8 Å), the λ values are close to zero, (i.e. these contacts are turned off), consistent with idea that restraints are turned off when they cannot be satisfied by the geometry of the protein. Interestingly, however, when the RMSD is lower (≈ 3 Å), the λ-values tend to fluctuate between one and zero. To depict a more direct correlation between the RMSD and the λ-values, we show a 2D-histogram over the sum of the four λ-values for the true contacts vs. the RMSD (Fig. S7). We find that low RMSD (≈3 Å) correlates with higher $\sum_i^4 \lambda_i$ which is consistent with the idea that given a fairly accurate structure, the true contacts are more likely to be turned on.

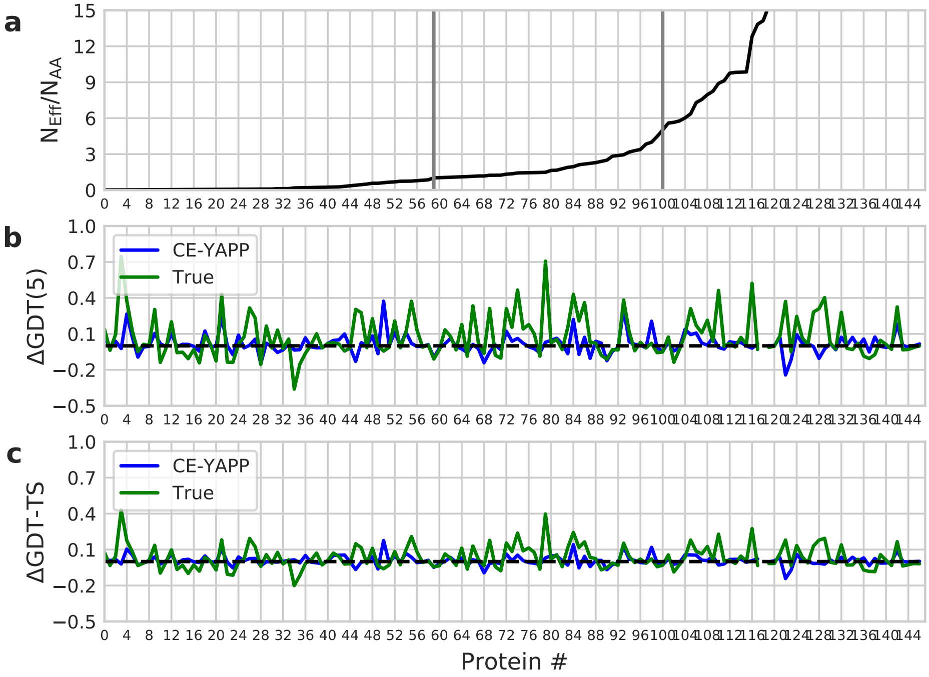

Figure S1: **Structural Performance on the NOUMENON dataset using Canonical Dihedral Angles.** These plots show the results of an equivalent analysis to that shown in Fig. 4 in the main text, but in this case using canonical dihedral angles for secondary structural elements predicted by PSIPRED.(a) The number of effective sequences divided by the number of amino acids, N_{Eff}/N_{AA}, is plotted for each protein and sorted from low to high. The data in the remaining panels are sorted accordingly. The grey vertical bars represent the proteins with N_{Eff}/N_{AA} closest to 1 and 5, respectively. (b) Difference in GDT(5) ($\Delta GDT(5)$). (c) Difference in GDT-TS (Δ GDT-TS). The black dashed line denotes zero.

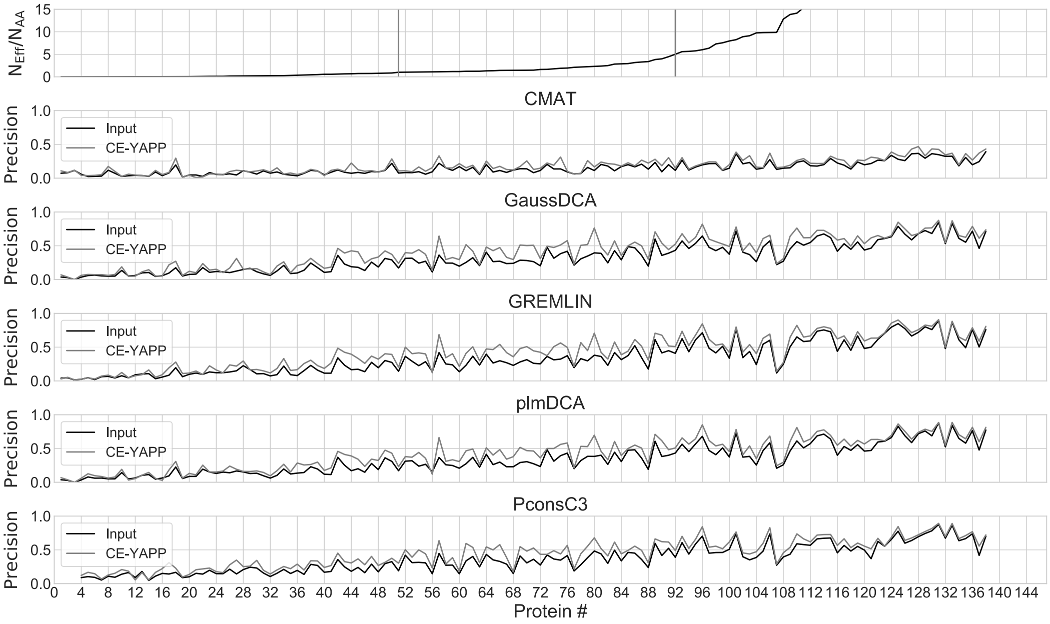

Figure S2: **Precision of the Contact Predictors.** Panel 1. The number of effective sequences divided by the number of amino acids, N_{Eff}/N_{AA}, is plotted for each protein, of the Noumenon data set, and sorted from low to high. The data in the remaining panels are sorted accordingly. The grey vertical bars represent the proteins with N_{Eff}/N_{AA} closest to 1 and 5, respectively. The remaining panels depict the precision (TP/(TP+FP)) of the input contacts and after applying CE-YAPP.

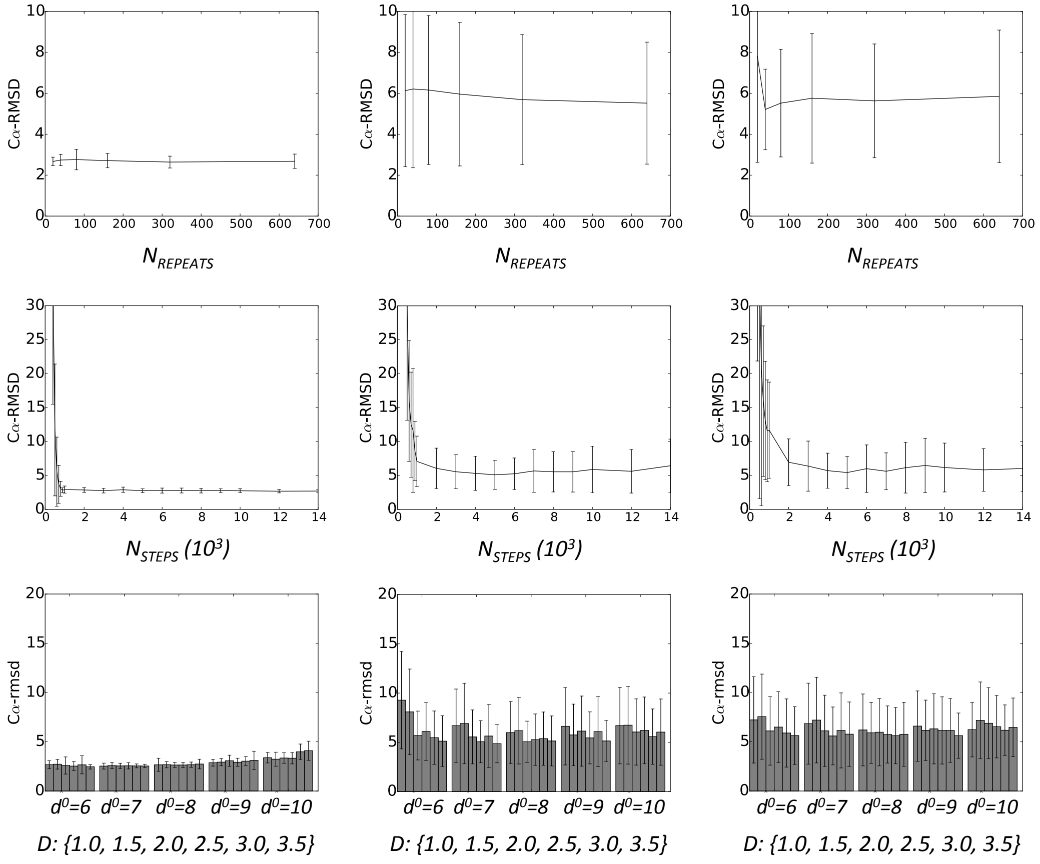

Figure S3: **Parameter Sweep.** Mean Cα-rmsd for 50 repeated simulations of three proteins (PDBID: 2RQL, 5P21, 1SVN) is plotted with respect to the parameters $N_{repeats}$ (top row), N_{steps} (middle row), D and d^0 (bottom row). Each bar plotted in the bottom row represents the mean Cα-rmsd for 50 repeated simulations with a specific d^0 and D (Eq. 3 in main text). All error bars represent the standard deviation for 50 repeated simulations.

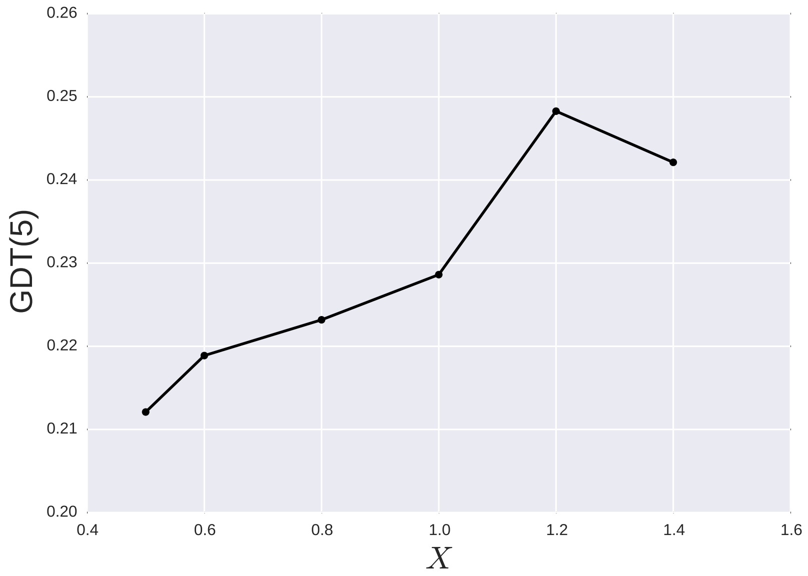

Figure S4: **Number of Input Contacts.** Total mean GDT(5) across 16 repeated simulations and each of the proteins in the Noumenon data set is plotted as a function of X, described in Eq. 1 in the Supporting Information.

a

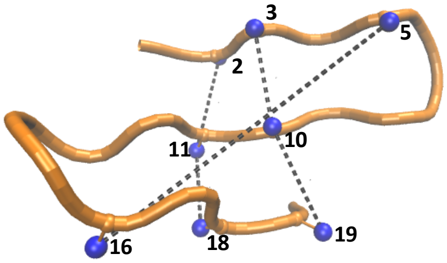

b

#	4	8	12	16	20
Seq.	TWIQ	NGST	KWYQ	NGST	KIYT
SS.	CEEE	ECCE	EEEE	ECCE	EEEC

Figure S5: **GSGS Structure and Contacts.** (a) Native structure of the GSGS peptide. The blue spheres represent Cβ atoms. The numbers indicate residue numbers and the black dashed lines connect the atoms for which we apply restraints. The contact/restraint between residue 5 and 16 is false, whereas the remaining contacts are true. (b) Sequence and secondary structure. Here, "C" denotes coil and "E" denotes extended strand. Only coiled amino acids have allowed degrees of freedom.

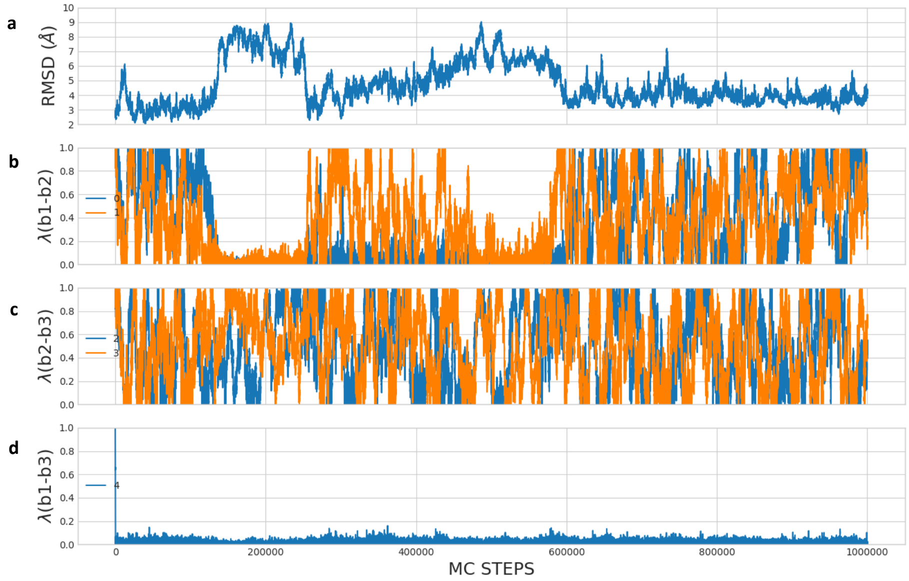

Figure S6: **Monte Carlo Trajectory** (a) RMSD. (b) λ-values for restraints between strand one and two. (c) λ-values for restraints between strand two and three. (d) λ-values for restraint between strand one and three.

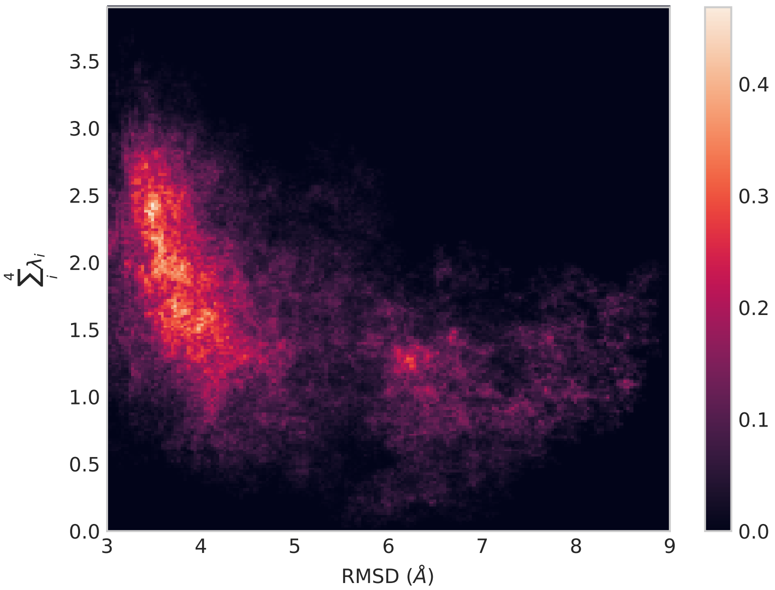

Figure S7: λ **vs. RMSD.** 2D histogram of the sum of the λ-values of the four restraints between strand 1&2 and strand 2&3 vs. RMSD. The colorbar reflects the probability.

References

[1] Ekeberg M, Lövkvist C, Lan Y, Weigt M, Aurell E. Improved contact prediction in proteins: Using pseudolikelihoods to infer Potts models. Phys Rev E. 2013;87:012707.

[2] Ekeberg M, Hartonen T, Aurell E. Fast pseudolikelihood maximization for direct-coupling analysis of protein structure from many homologous amino-acid sequences. Journal of Computational Physics. 2014;276:341 – 356.

[3] Baldassi C, Zamparo M, Feinauer C, Procaccini A, Zecchina R, Weigt M, et al. Fast and Accurate Multivariate Gaussian Modeling of Protein Families: Predicting Residue Contacts and Protein-Interaction Partners. PLOS ONE. 2014;9:e92721.

[4] Jeong CS, Kim D. Reliable and robust detection of coevolving protein residues. Protein Engineering, Design and Selection. 2012;25:705–713.

[5] Feinauer C, Skwark MJ, Pagnani A, Aurell E. Improving Contact Prediction along Three Dimensions. PLOS Computational Biology. 2014;10:e1003847.

[6] Wang S, Sun S, Li Z, Zhang R, Xu J. Accurate De Novo Prediction of Protein Contact Map by Ultra-Deep Learning Model. PLOS Computational Biology. 2017;13:e1005324.

[7] Cavalli A, Vendruscolo M. Analysis of the performance of the CHESHIRE and YAPP methods at CASD-NMR round 3. J Biomol NMR. 2015;62:503–509.
